# Intelligent Resource Allocation Scheme Using Reinforcement Learning for Efficient Data Transmission in VANET

**DOI:** 10.3390/s24092753

**Published:** 2024-04-26

**Authors:** Jin-Woo Kim, Jae-Wan Kim, Jaeho Lee

**Affiliations:** 1 Department of Statistics, Duksung Women’s University, Seoul 01369, Republic of Korea; jjin300@duksung.ac.kr; 2 School of Semiconductor & Electronic Engineering, Yeungjin University, Daegu 41527, Republic of Korea; jwkim@yju.ac.kr; 3 Department of Software, Duksung Women’s University, Seoul 01369, Republic of Korea

**Keywords:** VANET, reinforcement learning, Q-learning, IEEE 802.11p, WAVE, resource allocation

## Abstract

Vehicular ad hoc networks (VANETs) use multiple channels to communicate using wireless access in vehicular environment (WAVE) standards to provide a variety of vehicle-related applications. The current IEEE 802.11p WAVE communication channel structure is composed of one control channel (CCH) and several service channels (SCHs). SCHs are used for non-safety data transmission, while the CCH is used for broadcasting beacons, control, and safety. WAVE devices transmit data that alternate between CCHs and SCHs, and each channel is active for a duration called the CCH interval (CCHI) and SCH interval (SCHI), respectively. Currently, both intervals are fixed at 50 ms. However, fixed-length intervals cannot effectively respond to dynamically changing traffic loads. Additionally, when many vehicles are simultaneously using the limited channel resources for data transmission, the network performance significantly degrades due to numerous packet collisions. Herein, we propose an adaptive resource allocation technique for efficient data transmission. The technique dynamically adjusts the SCHI and CCHI to improve network performance. Moreover, to reduce data collisions and optimize the network’s backoff distribution, the proposed scheme applies reinforcement learning (RL) to provide an intelligent channel access algorithm. The simulation results demonstrate that the proposed scheme can ensure high throughputs and low transmission delays.

## 1. Introduction

Vehicular ad hoc networks (VANETs) are composed of multiple vehicles using wireless technologies to connect autonomously to the network, either vehicle-to-vehicle (V2V) or vehicle-to-infrastructure (V2I) installed near roads. VANET is a key component of intelligent transportation systems (ITSs) as it can provide safety services and various multimedia services to drivers, passengers, and even pedestrians. Dedicated short-range communication (DSRC) is a wireless communication protocol developed for toll collection systems. DSRC typically uses the 5 GHz band and has data transfer speeds exceeding several hundred kbps. However, DSRC standards are severely limited, and next-generation communication protocols suitable for VANET had to be to be developed. To achieve this, the IEEE established a task group (Task Group p) to enhance existing IEEE 802.11 standards to meet the requirements for WAVE [1]. Earlier, the American Society for Testing and Materials formulated ASTM E2213-03 which defines a communication protocol suitable for vehicular environments using orthogonal frequency division multiplexing (OFDM) in the 5.9 GHz RF band [2]. Consequently, Task Group p submitted an expanded IEEE 802.11p standard [1] which included WAVE and the innovations developed by ASTM E2213-03 [2].

IEEE 802.11p defines the medium access control (MAC) layer and the physical layer (PHY), considering the vehicular communication environment. While the original IEEE 802.11 required scanning, authentication, and association procedures for communication between nodes, IEEE 802.11p allows communication without these procedures using the outside context of a basic service set (OCB) mode.

IEEE 1609 defines the standards for the upper protocol layers of WAVE. IEEE 1609.1 defines higher-layer applications, IEEE 1609.2 outlines standards related to security services, and IEEE 1609.3 defines the wave short message protocol (WSMP) related to networking. Additionally, IEEE 1609.4 describes multi-channel operation. IEEE 1609.4 defines the control channel (CCH) and service channel (SCH), allocating different frequency channels to each and establishes the procedure for switching between these channels.

As a wireless transmission technology, WAVE supports data transfer speeds up to 27 Mbps in vehicles traveling at speeds up to 200 km/h. It introduces the concept of the WAVE basic service set (WBSS), considering the characteristics of vehicle networks, which is a distinct technical feature from the traditional IEEE 802.11 networks. The WAVE standard provides a multi-channel DSRC solution for various service types that can be used in VANETs. Based on this standard, many different services utilizing V2V and V2I communications are being developed. These services include collision warning services, traffic information and navigation updates, and infotainment

A WAVE communication system mainly consists of on-board units (OBUs) for vehicles and road-side units (RSUs). The OBU is installed inside the vehicle and connected to a terminal that interfaces with the user, providing services to drivers and passengers. The RSU is fixed along or near the road and offers connectivity to external networks. Therefore, the OBU can connect to external networks through the RSU.

WAVE communication systems can experience data loss and long data transmission times when there is a large volume of data to be transmitted over the CCH, due to the fixed CCH interval (CCHI). The static allocation of intervals for each channel can lead to specific channels being unnecessarily occupied without being operational, resulting in longer wait times. Additionally, other specific channels might consume a lot of time for data transmission, leading to delays in data transfer. In essence, the WAVE communication system is unable to efficiently utilize its limited channel resources.

Therefore, in this paper, we propose an adaptive resource allocation method for WAVE communication systems, which dynamically allocates channels by analyzing the amount of data traffic in both the CCH and SCH, allowing for the efficient use of limited wireless resources. To improve channel efficiency, the proposed scheme provides an intelligent resource allocation method that employs reinforcement learning for adjusting the contention window (CW).

The contributions of this paper are as follows:

- This paper presents an IEEE 802.11p protocol for dense scenarios.
- This paper studies the impact of the number of vehicle nodes on network performance in dense scenarios.
- A new adaptive resource allocation algorithm is proposed for allocating channel intervals based on vehicle density.
- A new CW (congestion window) adjustment algorithm using reinforcement learning (RL) to perform CW adaptation is proposed.
- The proposed algorithm enhances networking performance without significant modifications to existing hardware.

## 2. WAVE Protocols and Model

The WAVE physical layer is defined by IEEE 802.11p which is a modified form of existing wireless LAN standards, IEEE 802.11a/g. IEEE 802.11p uses frequencies ranging from 5.850 to 5.925 GHz, which is outside the industrial, scientific, and medical (ISM) band. Moreover, IEEE 802.11p utilizes OFDM to operate a single channel with a 10 MHz bandwidth. This reduction in channel bandwidth mitigates the effects of frequency-selective fading that often occurs at high speeds and in outdoor environments. Moreover, while existing wireless internet standards like IEEE 802.11a/g require an authentication process before establishing communication with an access point (AP), IEEE 802.11p/WAVE does not. This is because vehicles move at high speeds, and during the time required to authenticate communication between vehicles or between a vehicle and roadside infrastructure, the vehicle is likely to have passed. Therefore, IEEE 802.11p has a significantly faster communication setup, enabling vehicles to communicate instantaneously as long as the channel settings between vehicles or base stations are matched.

Omitting authentication procedures also make WAVE more secure and the WAVE IEEE 1609 standard has additional features that further enhance security. IEEE 1609 includes standards for V2V and V2I applications and provides resource management (RM) that ensures these applications use given resources efficiently (IEEE 1609.1). IEEE 1609 also enables safe communication over unsecured wireless networks (IEEE 1609.2), defines the network layer and transport layer (IEEE 1609.3), and enables multi-channel operation in the WAVE MAC layer (IEEE 1609.4).

**Figure 1:**
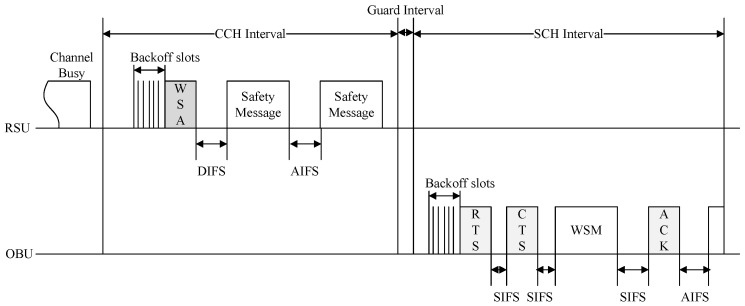
The WAVE standard channel structure.

IEEE 1609.4 defines the CCH and the SCH, allocating different frequency channels to each, and outlines the procedure for repeatedly switching between these channels. Figure 1 illustrates the WAVE channel structure.

The WAVE standard uses one CCH and six SCHs. The CCH is dedicated to system control and safety-related messages, while the SCHs are used for exchanging data packets not related to safety. The CCHI and SCH interval (SCHI) are both 50 ms in duration. The CCHI represents the duration of the CCH period, during which management messages for service advertisement and data messages of high-priority applications are transmitted. The SCHI represents the duration of the SCH period, during which typical data service messages are transmitted. During each CCHI, WAVE OBUs stay on the CCH to receive WAVE control packets and safety-related messages. During the SCHI, non-safety data are transmitted or received on the SCHs. Channel access for both the control and service channels follows the enhanced distributed channel access (EDCA) method defined by IEEE 802.11e.

The WBSS WAVE basic service set (WBSS) consists of a provider, who initiates the WBSS and other WBSS users who join it. The provider periodically transmits a beacon-like WAVE service announcement (WSA) containing network parameters such as information for identifying the WAVE application on the CCH, a WBSS identifier needed to join the WBSS, the SCH that the WBSS will use, and timing information for synchronization. Users can simply join the WBSS by receiving the transmitted WSA and then switching channels to the SCH used by the WBSS in the next SCHI. In this way, users can complete their transmission preparation without going through the subscription and authentication processes of IEEE 802.11 by just receiving the WSA.

WAVE communication devices perform channel switching and alternate between the CCH and the SCH. Currently, timing information messages are used to synchronize the absolute time among WAVE devices. However, there is no existing procedure for exchanging channel adjustment parameters necessary for channel switching.

## 3. Review of Related Work

In [3], the authors proposed an algorithm to dynamically allocate CCH intervals for the smooth transmission of beacon and safety-related messages. This algorithm divides the CCH interval into three segments, the service announcement phase, beacon phase, and peering phase, to reduce message collisions in the CCH interval. In [4,5,6], the authors proposed the variable CCH interval (VCI) protocol to ensure the transmission of safety messages while enhancing the quality of service in the SCH interval. VCI also divides the CCH interval into a safety interval and WSA interval to reduce collision probability in the CCH interval. In [7], the authors proposed the dynamic CCH interval (DCI) protocol to dynamically adjust the channel intervals. This protocol adjusts the CCH/SCH interval based on the reservation probability distribution of service messages. In [8], the authors proposed the dynamic safety interval (DSI) protocol, an improvement of the VCI protocol. DSI considers the number of vehicles sharing the channel, taking hidden nodes into account, and adjusts the safety interval accordingly. In [9], the authors proposed a protocol using Markov analysis to optimize the transmission probability of safety and WSA messages and dynamically adjust the ratio of CCH and SCH intervals. In [10], the authors proposed a protocol to adjust the control channel (CCH) interval based on real-time statistics and predictions of various types of messages.

In [11], the authors proposed the AMCMAC-D (asynchronous multi-channel medium access control with distributed) protocol, which does not rely on the RSU’s beacon frame for SCH channel usage. This protocol provides quality of service (QoS) by allocating different numbers of time slots for each access category (AC). In [12], the authors proposed a coordinated multichannel medium access control (C-MAC) protocol, which provides contention-free broadcasting for safety messages through the coordination of roadside units (RSUs). This protocol allows RSUs to reserve the broadcast transmission order of safety messages in the CCH interval and adjust contention intervals in the SCH interval.

The IEEE 802.11p standard adopted the channel access approach of the IEEE 802.11e standard, which defines four access categories (ACs), to provide quality of service (QoS) [1,13]. In [13,14,15,16], the authors proposed protocols for dynamically adjusting the contention window (CW) to reduce data collisions in congested wireless LAN environments. In [14], the authors proposed an enhanced distributed coordination function (EDCF) protocol that dynamically adjusts the minimum contention window (CWmin) based on channel conditions. This protocol provisions the priority between access categories by adjusting the size of the CWmin according to application requirements and channel conditions. In [15], the authors investigated the optimal CWmin considering the competitive nature of devices for channel access in IEEE 802.11. In [16], the authors proposed the adaptive enhanced distributed coordination function (AEDCF) protocol, which adjusts the CW sizes of each traffic class to provide relative priorities, considering both application requirements and network conditions. In [17], the authors proposed the traffic-aware enhanced distributed coordination function (T-EDCF) protocol, which adjusts CW sizes considering traffic flows. In [18], the authors proposed the sliding CW (SCW) protocol, which adjusts CW sizes within predefined ranges for each AC.

In [19], the authors point out that the high collision rate of broadcast messages is one of the main causes affecting the performance of V2X communication. In [20], the authors analyzed the broadcast performance of the control channel (CCH) in the IEEE 802.11p/WAVE standard and demonstrated that the number of vehicles and the size of the contention window (CW) impact the packet delivery rate. In [21], the authors showed that increasing the CW size can reduce the packet collision probability in dense traffic environments. In [22], the authors demonstrated that increasing the CW size can increase the packet transmission rate but also increases the delay. In [23], the authors proposed a swarming protocol to optimize one-hop delay by adjusting the CWmin of backup vehicles in multi-platoon scenarios.

In [24], the authors defined the backoff mechanism as a Markov decision process (MDP) and proposed a reinforcement learning algorithm to learn the backoff mechanism. In [25], the authors proposed a protocol applying a reinforcement learning framework to sensor networks, allowing sensor nodes to use optimal duty cycles by estimating the states of other nodes. In [26], the authors proposed a protocol using the Q-learning algorithm to adjust the contention window size.

The algorithms proposed in [3,4,5,6,7,8,9,10] simply double the size of the contention window (CW) if a packet collision occurs and reduce it to the minimum CW size if the transmission is successful. This method is inefficient because it determines the size of the CW based solely on whether a collision occurs, regardless of the number of wireless terminals in the network. If the size of the CW becomes excessively large compared to the number of terminals in the network, the probability of a successful transmission increases, but it also results in many wasted slots. After a successful transmission, the size of the CW is set to CWmin regardless of the number of terminals, and because it is based on the random backoff algorithm, the probability of collisions significantly increases in environments with high traffic or a large number of terminals.

The algorithms proposed in [11,12] have improved the idle state of the SCH, but because they use a fixed CCHI and SCHI, the CCHI remains idle for a significant amount of time. Conversely, the 40 ms length of SCHI is not sufficient when transmitting a large volume of non-safety messages, resulting in reduced throughput. Additionally, the IEEE 1609.4 vehicular network experiences a significant increase in the probability of collisions and reduced reliability in high vehicle density situations.

The algorithms proposed in [13,14,15,16,17,18] have implemented an active CW adjustment technique in WiFi, taking into account the channel status and network environment. However, in vehicular services, the duration of a single superframe interval is much shorter than what has been set in previous research, leading to high variability within individual SCHIs and making it difficult to infer reliable CW ranges for the average interval. This complexity makes collision estimation challenging. Moreover, individual frame sizes according to the service channel must be considered. As the data size increases and competition becomes more complex, as in this study, it becomes harder to manage these factors. Especially with very short SCHI times for each channel, there needs to be mechanism for adjusting CW based on the SCHI.

In [19,20,21,22,23], authors studied the relationship between the size of the contention window (CW), packet collisions, and delays. They conclude that there cannot be a one-size-fits-all CW value for all situations.

In [24,25,26], authors proposed algorithms that apply reinforcement learning to setting the size of the contention window (CW). However, this work does not consider the broadcast nature of VANETs, which transmit data within fixed channel intervals. Therefore, these algorithms do not address the issues of being unable to send data during limited times in dense vehicular environments and the increased probability of collisions. As a solution to the abovementioned limitations, we propose a Q-learning-based IEEE 802.11p MAC algorithm. This technique adaptively allocates channel resources, enhancing reliable packet delivery and throughput, and is sensitive to delays. Additionally, it increases reliability by using implicit acknowledgments (ACKs) for broadcasted messages.

## 4. Proposed Scheme

### 4.1. Basic Design

**Figure 2:**
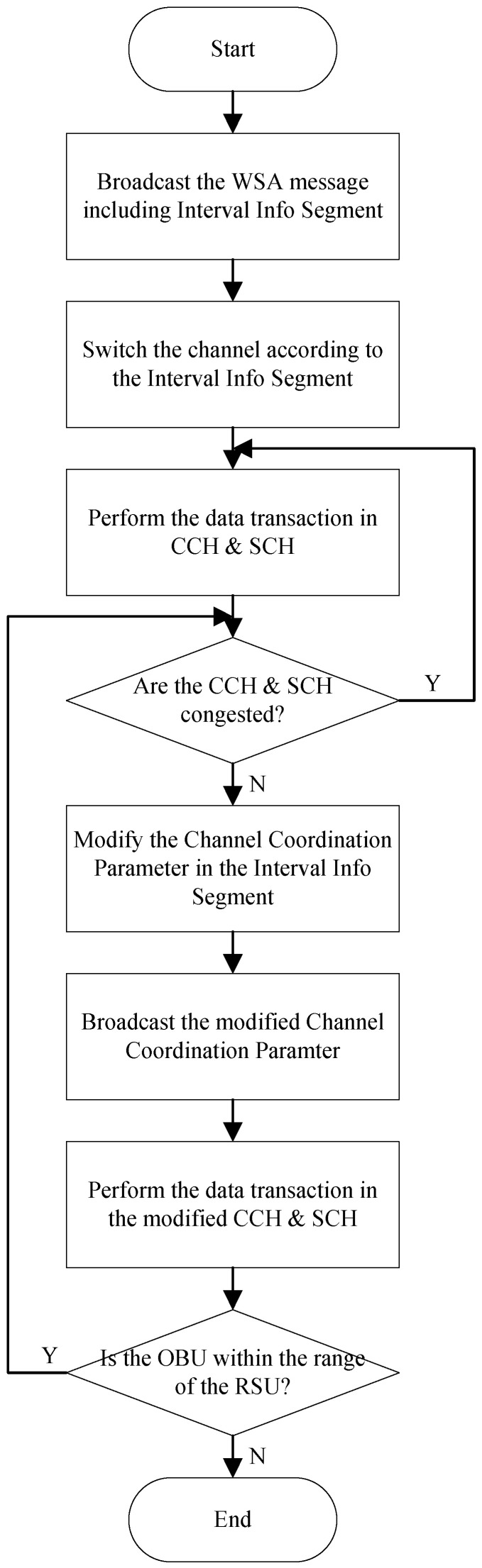
The proposed adaptive channel allocation scheme for WAVE.

Figure 2 shows the proposed adaptive channel allocation scheme for WAVE. RSUs periodically transmit WSA messages containing information about the services they offer through the control channel. In this process, RSUs include channel adjustment parameters in the WSA encompassing the CCHI, SCHI, guard interval length information (guard interval), and update time information (update time).

Before connecting to a specific RSU, OBUs in vehicles continuously monitor the control channel. By doing this, they can receive advertisement messages via the control channel upon entering the broadcast range of a specific RSU. After receiving the advertisement message, the OBU undergoes a synchronization process with the RSU. Subsequently, it begins channel switching between the control and service channels based on the channel adjustment parameters.

RSUs and OBUs in vehicles exchange data through the control channel and service channel while performing channel switching. During this data exchange, RSUs monitor the congestion level of the current CCH and SCH based on the amount of data traffic being exchanged. The congestion level of the channels, according to the volume of data traffic, can be determined by considering the average queuing delay of packets transmitted on each of the control and service channels at the MAC level, as well as the number of transmission failures due to data collisions.

If the monitored congestion level of a channel is higher on either the CCH or the SCH, the channel coordination parameter is adjusted to increase the interval length of the more congested channel and decrease the interval length of the less congested channel. However, since the CCH is more crucial due to the transmission of management messages like service advertisements and timing information, as well as high-priority messages, its interval length is adjusted to be above a minimum interval threshold, ensuring its essential functions are maintained.

The modified channel coordination parameters are transmitted from the RSU to the OBU. Channel switching is adjusted according to these adjusted channel coordination parameters, and data exchange continues through the adjusted CCH and SCHs. Monitoring the effectiveness of this data exchange, as well as the congestion levels based on the amount of data traffic in the CCH and SCHs, continues as long as the OBU remains within the broadcast range of the RSU.

### 4.2. The Proposed Adaptive Resource Allocation Scheme

An adaptive channel coordination scheme to improve the transmission efficiency of messages is proposed. Firstly, if a device intends to use services during the SCHI, it broadcasts information about the service during the CCHI through WSA messages. At this time, the device uses the existing extended field of the WSA message to indicate the data size of the service. Additionally, when allocating the SCH, all stations use an SCH allocation table to prevent service data from being biased towards one side. The SCH allocation table records the total expected service data for each SCH by examining WSA messages. The SCH allocation table is as shown in Table 1.

When constructing the WSA message, service providers check the SCH allocation table for each SCH number and the total amount of expected infotainment data. In Table 1, the service provider selects SCH 2, which has the smallest value, and adds the data size of this service to the total expected service data in their SCH allocation table. Other OBUs that receive this WSA message check the extension and service information fields of the WSA message to add the service data size to their own SCH allocation table. If these OBUs wish to use this service during the next SCHI, they add an ACK message to the next beacon message. This ACK message must include information such as user priority and data size if the OBU intends to send some data using this service during the next SCHI. The information includes (i) the number of stations participating in channel contention during the next SCHI, (ii) the provider service identifier (PSID) to identify the type of infotainment service, and (iii) the service priority field in the service field of the WSA message received during the CCHI.

The RSU uses the information to calculate the length of the transmission opportunity (TXOP) and the values for the next CCHI and SCHI and CW_min_ at the end of the CCHI. These modified values are then broadcast before the end of this CCHI.

The TXOP limit value is calculated as the total amount of service data divided by the number of stations that sent ACK messages. The length of the next CCHI and SCHI can be estimated based on the current amount of safety-related data traffic and the expected amount of infotainment data traffic. The amount of current safety-related data traffic, TD_CCH_, is calculated as follows [27]:

(1) $${\mathrm{TD}}_{\mathrm{CCH}}=\alpha \cdot E\left\{L\right\}\cdot \lambda ,$$

Here, E{L} is the average length of a safety packet, λ is the transmission frequency of safety packets, and α is a weight that considers the additional time due to channel collisions and the transmission of WSA packets.

Additionally, the estimated amount of infotainment data traffic is nearly equal to the maximum of the total estimated infotainment data for each AC in the RSU’s SCH allocation table at the end of the current CCHI. Therefore, the next lengths of the CCHI and SCHI, T_CCH_ and T_SCH_, respectively, are adaptively adjusted as follows:

(2) $$T_{\mathrm{CCH}}=\beta \cdot 100\cdot \frac{{\mathrm{TD}}_{\mathrm{CCH}}}{{\mathrm{TD}}_{\mathrm{SCH}}}\;T_{\mathrm{SCH}}=100-T_{\mathrm{CCH}},$$

Here, TD_SCH_ is the expected amount of infotainment data traffic and β is a weighting factor for safe driving. The loss of safety-related messages can have a critical impact on driver and passenger safety. Therefore, the value of β is predefined to be greater than 1.

To maintain the advantages of competitive channel access while reducing service data collisions and increasing service data throughput, CW_min_ is adjusted according to the number of service providers and the type of infotainment service offered by the RSU. When assigning the adaptive CW_min_ value, the RSU allocates the optimal CW_min_ in terms of total throughput. The method for optimizing CW_min_ is explained in detail in the following section.

### 4.3. The Proposed CW Adjustment Scheme Using Q-Learning

**Figure 3:**
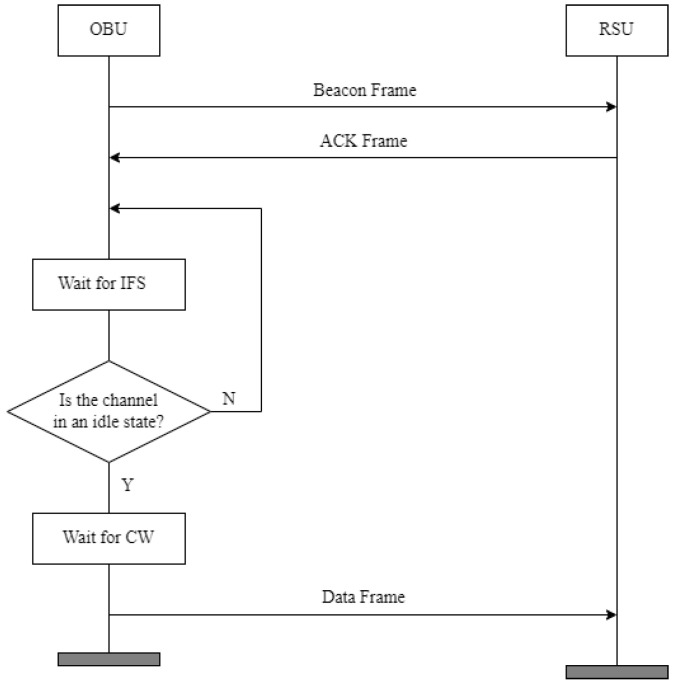
The timing diagram for the connection configuration between RSU and OBU.

Figure 3 shows the timing diagram for the connection configuration between the RSU and OBU. The OBU sends a beacon message to the RSU that includes the OBU’s physical and logical information, and the RSU uses this beacon message to initiate authentication and registration processes. The RSU then sends a response to the beacon message.

Upon receiving a response from the RSU, the OBU waits for an interframe space (IFS) duration. Afterward, the OBU checks if the channel between the RSU and OBU is idle. If the channel is not idle, the OBU waits further during the IFS time.

If the channel between the RSU and OBU is idle, the OBU waits for a period within the CW range before transmitting packet data to the RSU. This process helps prevent collisions that could occur when multiple OBUs transmit data simultaneously. The size of the CW must satisfy the following conditions:

- It should not increase packet transmission delays.
- It should not be unnecessarily large to avoid missing TXOPs.
- It should require minimal computational power so as not to strain the processor.

The CSMA/CA mechanism used in existing IEEE 802.11p and IEEE 1609.4 standards sets the channel access time of multiple devices randomly to avoid overlapping and thus prevent collisions. Therefore, each device maintains a small CW. However, recent studies show that setting a small CW is a major cause of packet collisions in the IEEE 802.11p network [28,29].

IEEE 802.11p and IEEE 1609.4 communication protocols manage vehicles broadcasting beacon and safety messages in the CCH interval and use the backoff algorithm to reduce collisions. In the case of broadcast communication, there is no way to check for collisions, so regardless of the success of a transmission, a fixed CW_min_ must be used, and a random value is selected from [0, CW_min_], followed by a wait for that duration (backoff delay) before broadcasting. Therefore, in a broadcast communication environment, when multiple nodes transmit data simultaneously, frequent transmission failures can occur due to collisions, necessitating the need to adjust the size of the CW to reduce the number of collisions and improve the reception success rate. The adaptive adjustment of the CW in the CCH using broadcast communication produces better performance than the existing method.

**Figure 4:**
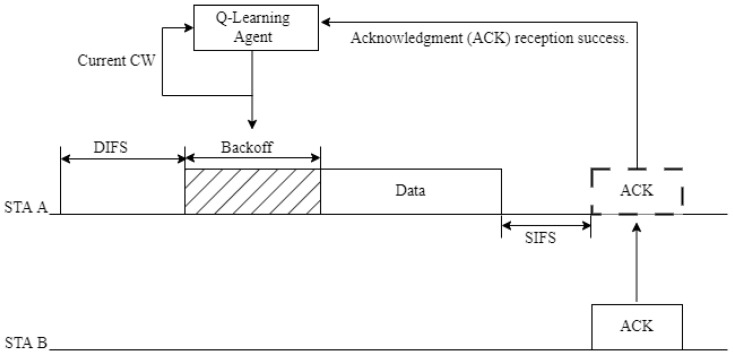
Proposed CW adjustment process.

**Figure 5:**
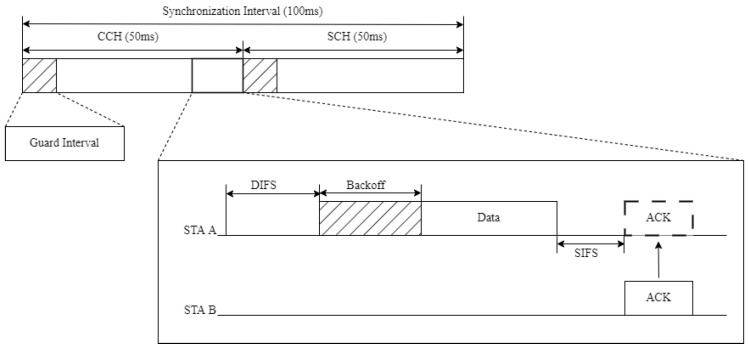
Proposed channel structure.

To address the abovementioned challenges, this paper proposes a reinforcement learning (RL)-based channel access technique that enables the efficient exchange of data packets among a large number of vehicles. Figure 4 illustrates the structure of the proposed CW adjustment technique, and Figure 5 shows the proposed channel structure.

In the CCH, broadcast communication is performed and, in this case, ACK messages are not received. Therefore, in the proposed technique, the frame structure of the WAVE standard can include a message exchange interval (an interval for receiving ACKs).

As depicted in Figure 5, at the end of the CCHI, the frame structure can be modified to include an interval for unicast communication in the CCH for a duration of T_CSV_ (CW suitability verification interval, CSV interval). During this time, each station (STA) can select one of its neighboring STAs to send and receive data (current CW check packet). An STA represents a device performing communication.

In the proposed scheme, the initial CW can be set to CW_min_. If STA A does not receive an ACK, instead of requesting retransmission of the ACK, STA A considers it a transmission failure. If STA A receives an ACK, it considers the transmission successful in the current contention window.

The CSV interval is determined based on Equation (3).

(3) $$T_{\mathrm{CSV}}=T_{\mathrm{DIFS}}+{\mathrm{CW}}_{\max}\times \mathrm{Slottime}+E\left[T_{\mathrm{Data}}\right]+T_{\mathrm{SIFS}}+E\left[T_{\mathrm{ACK}}\right],$$

In this context, T_DIFS_ represents the DCF interframe space time, CW_max_ is the maximum CW value, and Slottime is the time for a slot as defined in IEEE 802.11p (for example, the slottime for OFDM PHY defined in the IEEE 802.11p standard is 9 μs). E[T_Data_] is the expected time taken to transmit data (i.e., the current CW check packet) used in the CSV interval. T_SIFS_ is the short interframe space time, and E[T_ACK_] is the expected time taken to receive an ACK. In the proposed scheme, E[T_Data_] = (size of data)/(data rate), and E[T_ACK_] = (size of ACK)/(data rate).

### 4.4. CW Update Scheme Using a Q-Learning Model

In the proposed technique, the CW can be updated based on a Q-learning model. The Q-learning model is designed using the following equation:

(4) $$Q\left(S_{t},a_{t}\right)\leftarrow \left(1-\alpha \right)Q\left(s_{t},a_{t}\right)+\alpha \left[r_{t+1}+\gamma \underset{a_{t+1}}{\max}Q\left(s_{t+1},a_{t+1}\right)\right],$$

where s_t_ represents the state (the size of the CW) at time t, a_t_ is the probability of action at time t, α is the learning rate, γ is the discount factor, and r_t_ is the reward value at time t. Specifically, s_t_ at time t could be 3, 7, 15, 31, 63, 127, or 255. In this case, CW_min_ could be 3. Moreover, a_t_ could involve actions to reduce, maintain, or increase the CW. For learning based on new information, α is close to 1. Meanwhile, γ as the discount factor can be close to 1 for learning towards higher rewards and close to 0 for learning towards current rewards. Additionally, r_t_ as the reward value at time t can be set to 1 if an ACK message is received and −1 if it is not received.

If the CW is at minimum, it can set the initial Q-table. Specifically, the first STA can set the CW during the initial broadcast communication process to CW_min_ (CW = 3). In this case, the Q-table includes rows 1 to 7 for the CW size and columns 1 to 3 for the backoff exponent (BE) and is used to decide the action for changing the CW size. Specifically, the initial Q-table representing the Q values for the CW in the aforementioned initial communication process is as shown in Table 2.

In the proposed scheme, CW can be updated based on the Q-learning model, with the BE being a parameter that decides the action for changing the size of the CW, as determined by Equation (5).

(5) $$BE=\log_{2}\left(Size\;of\;CW+1\right),$$

When the action determined by the Q-table is BE − 1, the first STA reduces the size of the contention window; if it is BE, the STA maintains the size; and if it is BE + 1, the STA increases the size. Moreover, as the contention window changes through the Q-learning model, the STA can update the Q-table as depicted in Table 2. In the proposed technique, with each update, there is a 0.9 probability of choosing the action corresponding to the column with the highest value among BE − 1, BE, and BE + 1 in the updated Q-table. There is also a 0.1 probability of randomly choosing any column corresponding to BE − 1, BE, or BE + 1.

By adding randomness to the change in the CW, the technique ensures dynamic adjustment. In cases where the updated Q-table has identical values for the row corresponding to the CW, the Q-learning model is designed to reduce the size of the contention window, thus preventing its expansion. For example, if the values in columns BE − 1, BE, and BE + 1 of the current state in the Q-table are all the same, the column corresponding to BE − 1 can be chosen as the action.

In cases where two of the three values in the updated Q-table are the same, and the third value is smaller, the STA can choose the action corresponding to the column of the two identical values. For example, if BE − 1 and the BE columns have the same values and BE + 1 is smaller, the column corresponding to BE − 1 can be chosen as the action.

The proposed scheme allows choosing an action that reduces the size of the CW among two identical actions. For instance, if the first largest value is in the BE − 1 column and the second largest value is in the BE + 1 column, and the difference between these values is within a pre-set range, the column corresponding to BE − 1 can be chosen as the action. Even if the values corresponding to actions are different, if the difference between the largest and the second or third largest values is minimal, the STA can set the action towards reducing the size of the contention window.

As an example, in the process of changing the contention window using the proposed technique when the initial state st_0_ is 3, the initial action at_0_ is BE, α is 0.6, and γ is 0.9 is examined. The initial state Q-table is as shown in Table 3, with the value corresponding to the current state being one-third (marked in red).

In this scenario, assume for example that the first STA (device) fails to receive an ACK message from a second STA (another device), and a probability of 0.9 is selected. This means that the current state s_t0_ is 3, and a_t0_ is BE (maintaining the current state), so s_t1_ could be a contention window size of 3 (maintaining the current state).

The Q-learning model can operate according to the following equation and update the Q-table accordingly:

(6) $$Q\left(s_{t1},\;a_{t1}\right)\leftarrow \left(0.4\right)Q\left(s_{t0},\;a_{t0}\right)+0.6\left[r_{t1}+0.9\underset{a}{\max}\left(s_{t1},\;a_{t1}\right)\right].$$

In this case, since the first STA fails to receive an ACK message, r_t+1_ = −1. Also, this corresponds to the value when the contention window is 3 and the action is in the BE column, which has a value of one-third.

Furthermore, $\left[\underset{a}{\max}Q\left(s_{t0},\;a\right)\right]$ is the maximum value of a for state st0, so it takes the value of one-third corresponding to the case when the CW is 3 and the action is in the BE column. Consequently, $Q\left(s_{t0},a_{t0}\right)=\left[0.4\times \frac{1}{3}+0.6\times \left[-1+0.9\times \frac{1}{3}\right]\right]=-0.28667$ (marked in red). Based on this, the updated Q-table is as shown in Table 4 below.

Since the largest value from the row with a CW of 3 is one-seventh (in the BE + 1 column), at_1_ can be chosen as the action corresponding to BE + 1 (i.e., an action to increase the CW size). The Q-table in the updated state is as shown in Table 5 below, and in this case, the value corresponding to the current state is one-seventh (marked in red).

In this scenario, assuming that the first STA (device) fails to receive an ACK message from the second STA (another device), and a probability of 0.9 is selected, the current state s_t1_ is 3, and at1 is BE + 1; hence, s_t2_ changes to a contention window size of 7. The Q-learning model operates according to Equation (7) and updates the Q-table shown in Table 5.

(7) $$Q\left(s_{t2},a_{t2}\right)\leftarrow 0.4Q\left(s_{t1},a_{t1}\right)+0.6\left[r_{t2}+0.9\underset{a}{\max}\left(s_{t2},a_{t2}\right)\right],$$

In this situation, since the first STA fails to receive an ACK message, r_t+1_ = −1. Also, $Q\left(s_{t1},a_{t1}\right)$ corresponds to the scenario where the CW is 3 and the action is in the BE + 1 column, which has a value of one-seventh. Furthermore, $\left[\underset{a}{\max}Q\left(s_{t2},a_{t2}\right)\right]$ is the maximum value of a for state s_t1_, so it takes the value of one-seventh, corresponding to the scenario where the CW is 3 and the action is in the BE + 1 column.

Consequently, $Q\left(s_{t2},a_{t2}\right)=\left[0.4\times \frac{1}{7}+0.6\times \left[-1+0.9\times \frac{1}{7}\right]\right]=-0.46571$ (marked in red). Based on this, the updated Q-table is as shown in Table 6 below.

Since the largest value for the row with a CW of 3 is −0.28667 (in the BE column), a_t2_ can be chosen as the action corresponding to BE (i.e., an action to maintain the CW size). Through this process, the Q-table is updated, and the size of the contention window can be changed according to the updated Q-table.

### 4.5. The Sync Interval Adjustment by RSU

**Figure 6:**
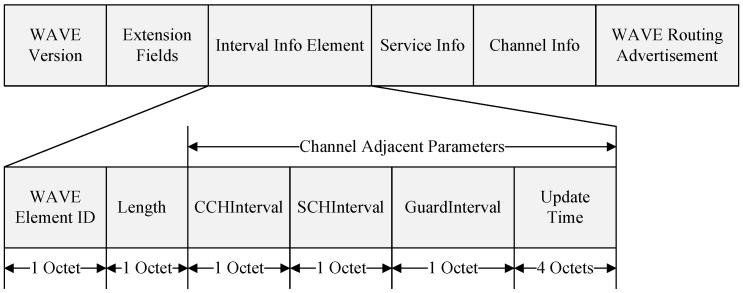
The proposed WSA frame.

Figure 6 shows the WSA frame. The advertisement message (WSA) includes a header, Interval Info Element, provider service table, and a WAVE routing advertisement. The header is defined in the WAVE standard and can include an optional extension field. The WAVE Version can differentiate messages by incrementing the ‘change count’ each time a message is transmitted. The interval info element contains the WAVE element ID, length, control channel length information (CCHInterval), service channel length information (SCHInterval), Guard Interval, and Update Time.

The WAVE Element ID is chosen from one of the areas allocated for future use in the WAVE communication standard and indicates the location of the Interval Info Element. The Length represents the length of the content included in the Interval Info Element, with the value of the Length field pointing to the length of the channel coordination parameter. The channel coordination parameter includes the CCHInterval, SCHInterval, Guard Interval, and Update Time.

The Update Time indicates the timing of channel switching according to the channel coordination parameter, specifying when (coordinated universal time, UTC) the channel switching should occur. For example, if the update time is ‘0’, channel switching occurs at the start of the next second of absolute time as per the channel coordination parameter.

**Figure 7:**
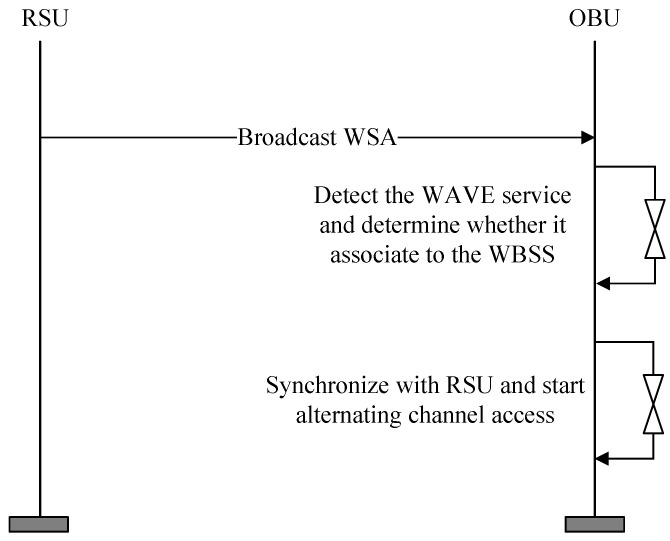
Data flow for the initial exchange of the channel coordination parameters.

The WAVE Routing Advertisement involves the RSU transmitting its IP address to the OBU in the vehicle, which can then use the received IP address. However, this is optional and can be omitted if an IP address for use between the OBU and RSU has been predetermined. Figure 7 shows a flowchart depicting the initial exchange process of the channel coordination parameters in the proposed scheme.

**Figure 8:**
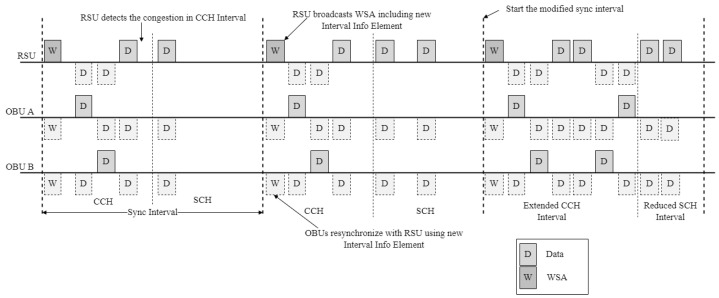
Representative data flow and time synchronization in the proposed scheme.

The RSU transmits an advertisement message to the OBU in the vehicle, which includes information about the services it offers and the channel coordination parameters. Upon receiving this advertisement message, the OBU decides whether to receive services from the RSU. If it decides to receive services, the OBU synchronizes its timing with the RSU. Subsequently, the RSU transmits advertisement messages and data through the CCH and SCH. While transmitting data, the RSU periodically monitors the real-time congestion levels of the CCH and SCH based on the amount of data traffic. If the congestion level is high in one channel, the RSU adjusts the channel coordination parameters to increase the interval length of the congested channel. The adjusted channel coordination parameters are included in a new interval information response message and transmitted to the OBU through the CCH. The OBU and the RSU then perform channel switching in accordance with the update time information of the adjusted channel coordination parameters. As a result, the length of the channel with high congestion is increased, and the length of the channel with low congestion is decreased. Figure 8 shows a flowchart depicting the process of changing the channel coordination parameters in the proposed intelligent resource allocation scheme.

In the scenario shown in Figure 8, the RSU detects high congestion in the CCH. The RSU creates a new interval information element to increase the length of the CCHI. Subsequently, it broadcasts the WSA containing the new interval information element. If the update time information of the adjusted channel coordination parameters is ‘0’, the adjusted channel coordination parameters are applied from the start of the next absolute time, increasing the length of the CCH and decreasing the length of the SCH for channel switching. The OBUs that receive the new interval information element resynchronize with the RSU and smoothly communicate during the extended CCH time interval.

### 4.6. Sync Interval Adjustment by OBU

**Figure 9:**
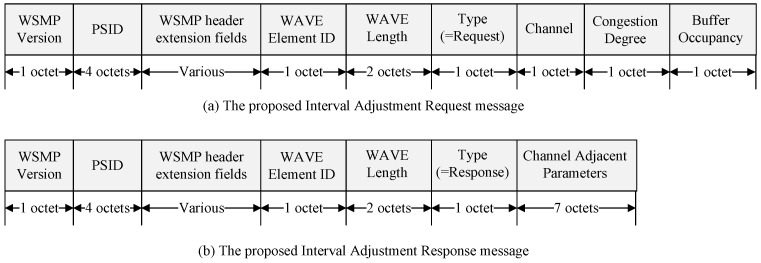
Formats of the proposed Interval Adjustment messages: request and response.

When an OBU detects congestion, it must also inform the RSU. During congestion, the OBU sends an Interval Adjustment Request message to the RSU. Upon receiving the Interval Adjustment Request message, the RSU generates channel coordination parameters, including CCHInterval, SCHInterval, Guard Interval, and Update Time. The RSU then broadcasts an Interval Adjustment Response message containing the new channel coordination parameters. The OBU, after receiving the response message, undergoes a synchronization process to align its timing with the RSU. Then, it begins channel switching between the control and service channels according to the received channel coordination parameters. Figure 9 shows the structure of the Interval Adjustment messages.

Figure 9a shows the proposed Interval Adjustment Request message. The WSMP version field indicates the WAVE protocol version. The PSID is a numerical field used in the IEEE 1609 standard, utilized for identifying specific applications. To access WAVE services, an application must be registered with a unique PSID. WAVE provider devices use the PSID in their announcement messages to indicate the provision of specific applications. The WSMP header extension field defines the channel used for communication and determines the length of the WSMP header, represented by the WAVE Element ID field and the data field. The Channel field indicates the channel where congestion occurred. The Congestion Degree field contains the congestion degree calculated by the device, and the Buffer Occupancy field includes the buffer occupancy calculated by the device.

**Figure 10:**
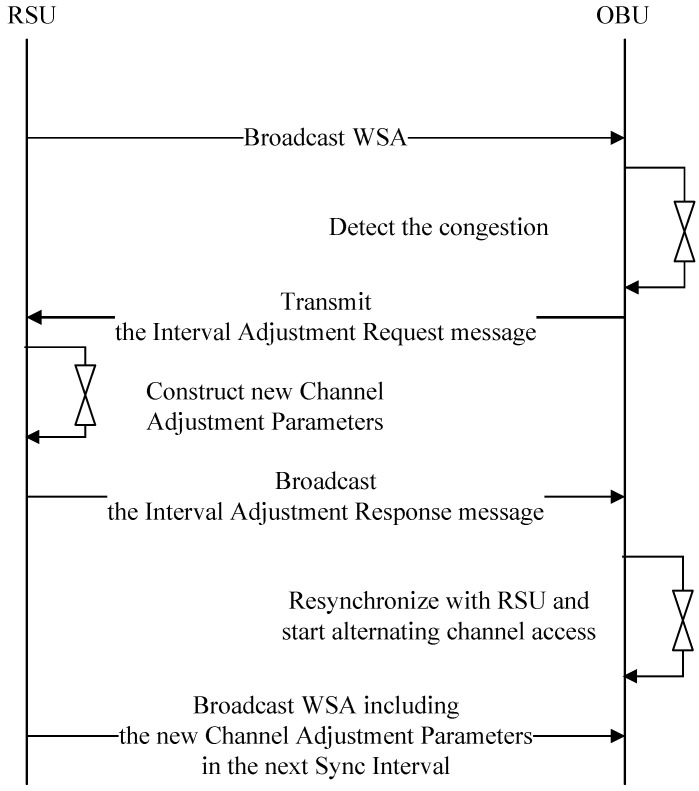
Timing diagram for Sync interval adjustment by the OBU.

Figure 9b displays the structure of the proposed Interval Adjustment Response message. The Type field indicates whether the interval information element is a request or a response, with the Type in the Interval Adjustment Response message corresponding to a response. The channel adjustment parameter field includes CCHInterval, SCHInterval, Guard Interval, and Update Time. The Update Time in the Interval Information Response message is ‘0’, so the channel coordination parameters are applied from the start of the next absolute time, enabling channel switching. Figure 10 shows a flowchart depicting the process of changing the Sync Interval when an OBU detects congestion.

**Figure 11:**
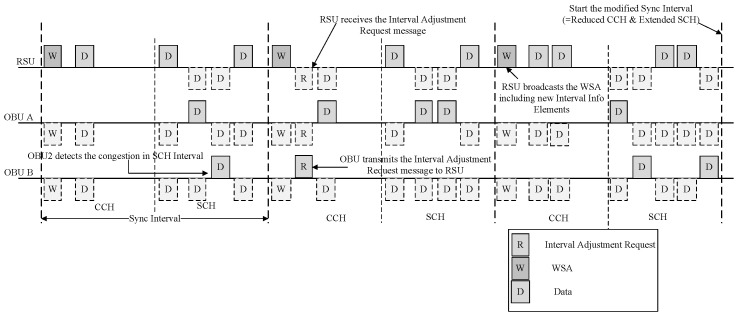
Representative data flow and time synchronization requested by an OBU.

The RSU transmits an advertisement message to the OBU in the vehicle, which includes information about the services it offers. The OBU, upon detecting congestion, sends an Interval Adjustment Request message to the RSU requesting channel adjustment. Upon receiving the Interval Adjustment Request message, the RSU broadcasts an Interval Adjustment Response message containing new channel coordination parameters. The OBU, after receiving the new channel coordination parameters from the RSU, performs re-synchronization. Starting from the next Sync Interval, the RSU broadcasts the WSA containing the new channel coordination parameters. Figure 11 shows a flowchart depicting the process of Sync Interval adjustment by the OBU.

In Figure 11, the OBU detects high congestion in the SCH. The OBU sends an Interval Adjustment Request message to the RSU in the CCH, requesting an increase in the SCHI. Upon receiving the Interval Adjustment Request message, the RSU modifies the Sync Interval and broadcasts an Interval Adjustment Response message containing the modified information. If the update time information of the modified channel coordination parameters is ‘0’, the OBUs that receive the Interval Adjustment Response message will apply the modified channel coordination parameters from the start of the next absolute time, decreasing the length of the CCH and increasing the length of the SCH for channel switching. The RSU will broadcast WSA containing the modified interval information elements from the next absolute time onwards.

## 5. Performance Evaluation

**Figure 12:**
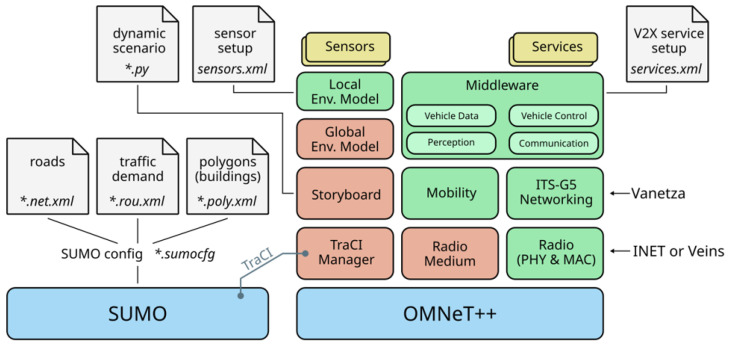
OMNeT++, SUMO, and Veins simulation framework.

**Figure 13:**
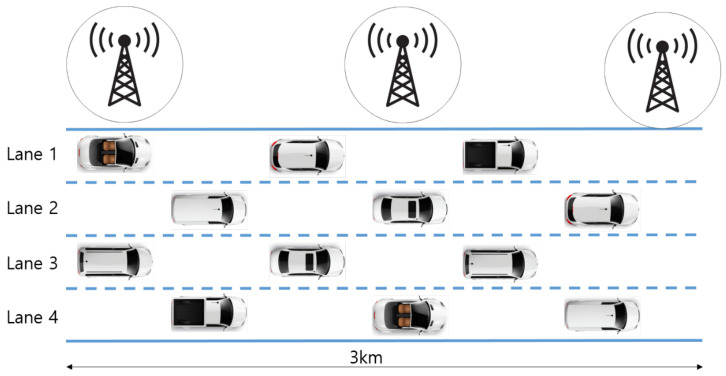
The simulation scenario.

Integrated simulation using OMNeT++ [30], SUMO (Simulation of Urban MObility) [31], and Veins (Vehicles in Network Simulation) [32], as shown in Figure 12, was performed. SUMO is a simulator focused on creating network scenarios and implementing mobility models. It is used to generate all roads and vehicles in the simulation of the proposed technique. OMNeT++ implements the IEEE 802.11p and WAVE standards and simulates the network. Veins connects OMNeT++ with SUMO. Figure 13 shows the highway traffic scenario utilized for the simulation. To evaluate the performance of the proposed technique, a 3 km four-lane highway was simulated. Table 7 shows the traffic parameters for the highway used in the simulation experiment. To simulate different levels of traffic congestion, various numbers of vehicles were considered. The number of vehicles is set at intervals of 20, ranging from 40 to 120 (five levels of congestion), with 120 vehicles corresponding to the vehicle density during rush hour on the highway [33]. As for traffic flow considerations, vehicles follow the Krauss model [34] with a maximum speed of 16 m/s. Furthermore, two conditions are implemented for the traffic scenarios: (1) All vehicles are within a one-hop communication range (approximately 1.2 km) [35], and their positions do not change. Under this condition, the hidden terminal problem is eliminated, allowing for the accurate measurement of packet collisions as the number of vehicles increases. (2) Since this simulation primarily focuses on improving communication performance with the increase in the number of vehicles within a one-hop communication range, obstacles like buildings are not considered.

In this simulation, the data transmission rate is set to 6 Mbps [36] with the primary objective of evaluating the proposed algorithm in a congested VANET based on the number of vehicles within a one-hop communication range. Higher data rates may limit the communication range, so the data rate should be adjusted according to the communication requirements of various safety applications. The configuration parameters for the evaluation are summarized in Table 7.

**Table 7:** Simulation parameters.

Parameter	Value
Channel Frequency	5.89 GHz
Channel Bandwidth	10 MHz
Number of vehicles	20–120
Transmission rate	6 Mbps
Transmission power	30 dBm
Safety packet size	256 bytes
Non-safety packet size	400 bytes
Packet generation frequency	10 Hz
Propagation model	Nakagami model [37]

**Figure 14:**
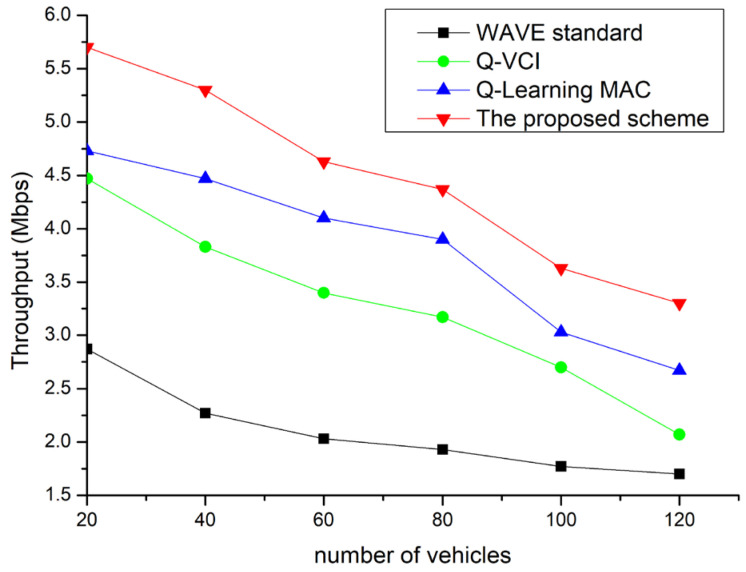
Throughput vs. network density for different VANET communication protocols.

Figure 14 shows the throughput of the proposed scheme compared to existing protocols based on the number of OBUs. As the number of OBUs increases, so does the number of collisions in the channel, leading to degraded performance in all protocols. In the case of the Q-VCI protocol [6], more time is allocated to the CCHI and less to the SCHI, whereas with the WAVE standard, competition for an SCH intensifies as the number of nodes increases. The Q-learning MAC [26] can mitigate throughput degradation by finding the optimal CW but has a lower throughput than the proposed technique as it transmits data in fixed channel intervals. The Q-VCI protocol, while providing variable channel intervals, has a lower throughput than the proposed scheme due to constraints on the CW. The proposed scheme demonstrates superior network performance by adaptively allocating channel resources and providing the optimal CW in congested network situations.

**Figure 15:**
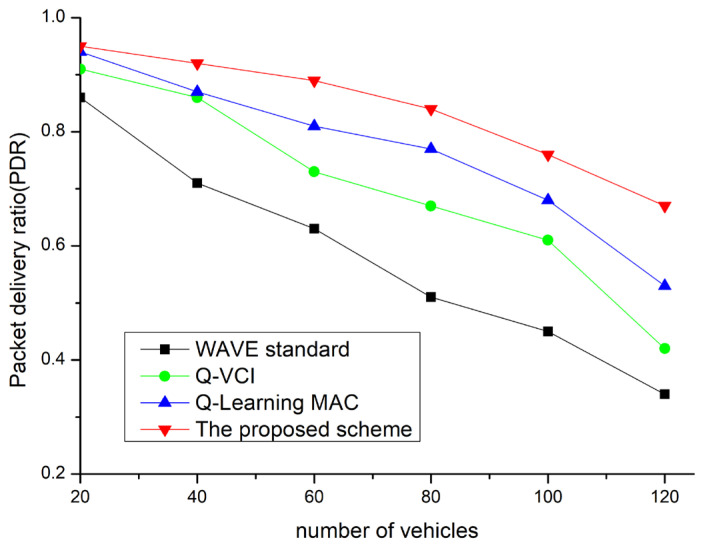
PDR vs. network density.

Figure 15 shows the packet delivery ratio (PDR) evaluation results according to the number of V2V interactions. In all cases, there is a downward trend as the number of vehicles increases, reflecting the fundamental resource constraints of VANETs. Despite the overall decline, the proposed technique maintains the highest average PDR in all scenarios, while the Q-learning MAC demonstrates a relatively poorer performance. With the WAVE standard, the PDR decreases in denser networks due to increased collisions between data packets. In the case of the Q-VCI protocol, the ability to adjust channel intervals mitigates PDR degradation. Additionally, the Q-learning-MAC, designed to adjust the size of CW as needed, can also reduce the decrease in PDR.

The proposed scheme, by adjusting channel intervals and finding the optimal CW through Q-learning, demonstrates significantly better performance in terms of successful data delivery. Furthermore, the ability to learn through ACK reception in the CCH interval allows CW optimization.

**Figure 16:**
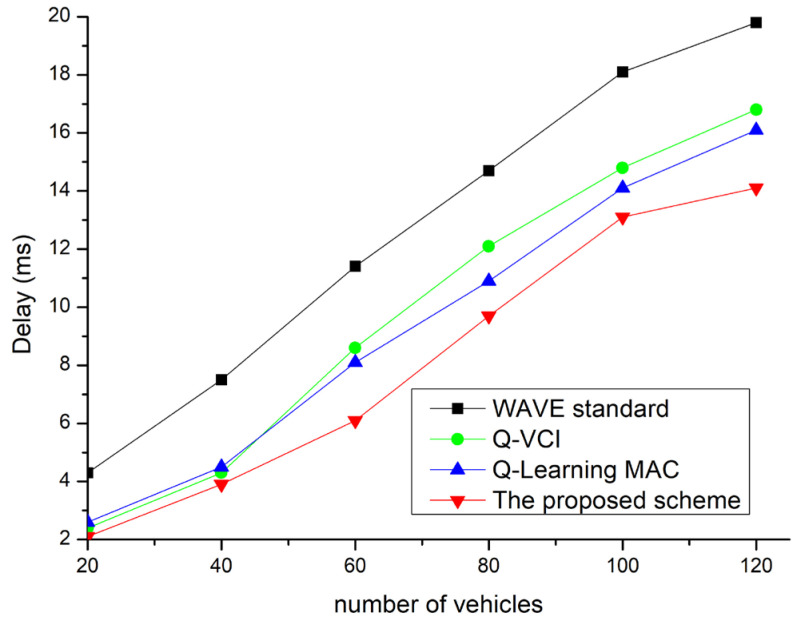
Delay vs. network density.

Figure 16 shows the average delay based on the number of vehicles. The delay worsens as the congestion level increases, which is due to more vehicles attempting channel access for safety broadcasts. The existing protocol exhibits the lowest delay values because successfully broadcasted packets are rare due to frequent packet collisions. The results reveal a clear trade-off between PDR and delay as a higher PDR leads to a longer delay in a congested VANET. To accommodate channel access without packet collisions in a congested network, the CW value must be sufficiently large, but a larger CW increases the delay. Therefore, adaptive algorithm selection is required based on different objectives and unique delay requirements for different applications. The proposed technique supports channel interval adjustments and exhibits lower delay times compared to other protocols.

**Figure 17:**
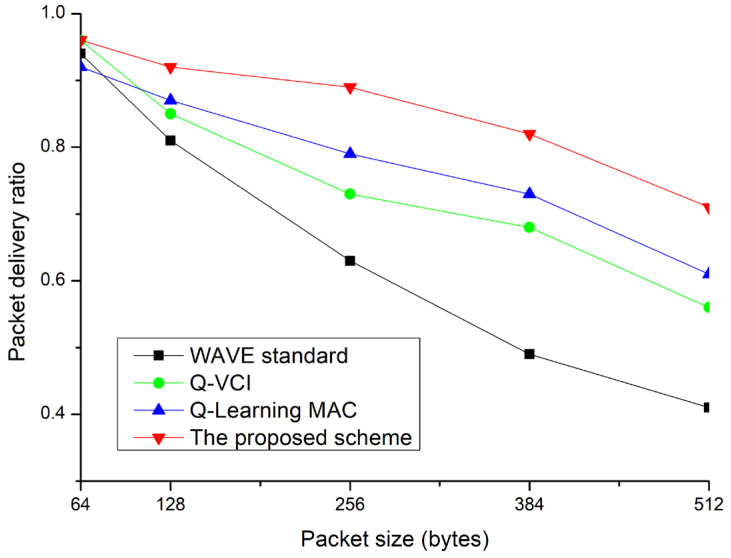
PDR vs. packet size.

Figure 17 illustrates the PDR performance based on packet size. As the packet size increases, it exacerbates network congestion. Consequently, all protocols exhibit a decrease in PDR as the packet size grows. However, even as network congestion worsens due to larger data exchanges, the proposed technique is more robust compared to other methods. Performance degradation due to network congestion can be compensated with higher data transmission rates. However, higher data rates limit the communication range, necessitating adjustments in data rates based on communication requirements.

**Figure 18:**
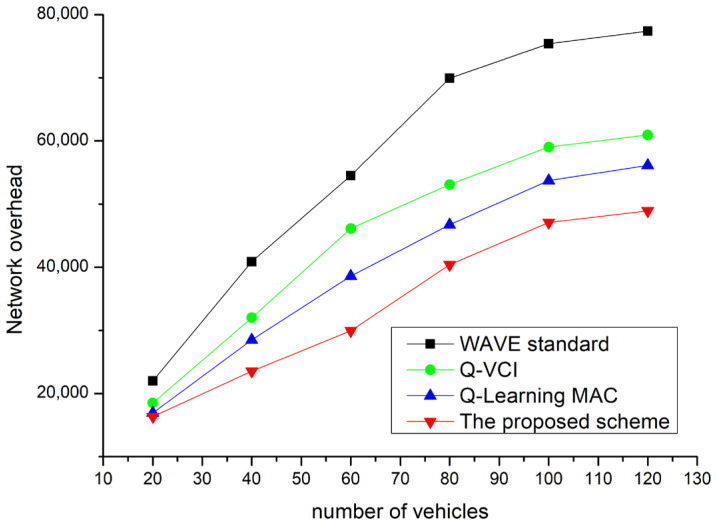
Network overhead vs. network density.

Figure 18 is a graph of the total network load. Since power is consumed in the packet transmission of nodes, it is advantageous in terms of power consumption to achieve higher throughput with fewer packet transmissions. As seen in the figure, it can be observed that network overhead increases as the number of nodes increases. This is due to the increase in the number of packets transmitted as the number of vehicles increases. The increase in packets with the increase in vehicle numbers is a natural phenomenon. However, the increase in retransmissions due to packet loss is also a major cause of increased overall network packet transmission. The proposed technique dynamically adjusts the CW and channel intervals to reduce the number of packet retransmissions caused by collisions. Since the proposed technique does not transmit separate control packets to adjust the CW, there is no increase in network overhead from this. Additionally, the technique uses existing WSA packets instead of sending separate control packets to adjust channel intervals. Therefore, this results in a lower network overhead compared to other protocols.

**Figure 19:**
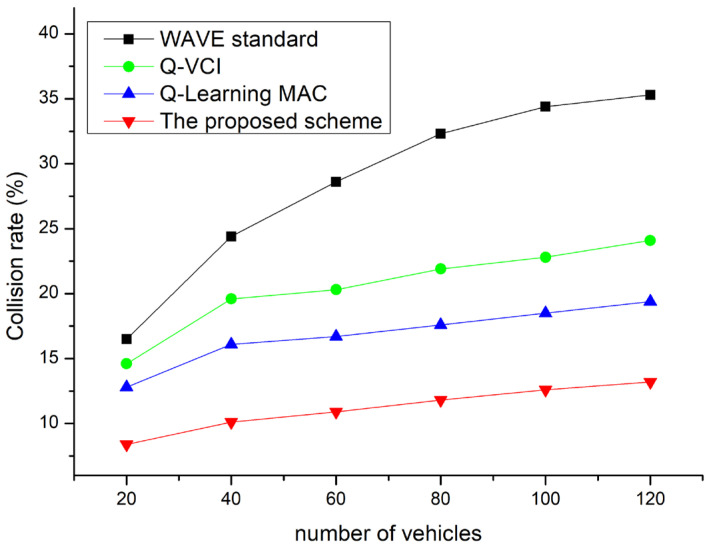
Probability of collisions vs. network density.

Figure 19 shows the probability of collisions according to the number of devices. As seen in the figure, the higher the number of vehicles, the higher the collision rate. In Figure 18, the Q-VCI and Q-learning MAC algorithms showed better performance than the WAVE standard. Compared to others, the proposed algorithm is efficient at drastically reducing the collision rate. The main reason is that the proposed technique can adaptively adjust the channel intervals and CW to reduce the collision rate when there are many competing stations. Therefore, the proposed technique can effectively reduce the collision rate.

## 6. Conclusions

In this paper, we proposed a method for adaptively adjusting channel intervals and the CW dynamically to enhance network performance in VANET. By optimizing the performance of the CCHI and SCHI, minimizing the CW, and applying TXOP limits based on predicted data traffic, higher throughput and lower latency can be achieved. Therefore, collisions in the channel can be mitigated. Through simulations, we verified that the proposed algorithm surpasses existing protocols in terms of throughput and delay. Thus, by using an adaptive channel adjustment approach, it is possible achieve stable transmissions while increasing the number of OBUs.

## Figures and Tables

**Table 1:** SCH assignment table.

SCH Number	Total Amount of Estimated Service Data (Byte)
1	1632
2	338
3	789
4	612
5	1266
6	2482

**Table 2:** Initial Q-table.

	BE − 1	BE	BE + 1
3	−100	1/3	1/7
7	1/3	1/7	1/15
15	1/7	1/15	1/31
31	1/15	1/31	1/63
63	1/31	1/63	1/127
127	1/63	1/127	1/255
255	1/127	1/255	−100

**Table 3:** Initial selection in the initial Q-table.

	BE − 1	BE	BE + 1
3	−100	1/3	1/7
7	1/3	1/7	1/15
15	1/7	1/15	1/31
31	1/15	1/31	1/63
63	1/31	1/63	1/127
127	1/63	1/127	1/255
255	1/127	1/255	−100

**Table 4:** Updated Q-table when failing to receive an ACK message.

	BE − 1	BE	BE + 1
3	−100	−0.28667	1/7
7	1/3	1/7	1/15
15	1/7	1/15	1/31
31	1/15	1/31	1/63
63	1/31	1/63	1/127
127	1/63	1/127	1/255
255	1/127	1/255	−100

**Table 5:** Updated Q-table when choosing an action to increase the CW size.

	BE − 1	BE	BE + 1
3	−100	−0.28667	1/7
7	1/3	1/7	1/15
15	1/7	1/15	1/31
31	1/15	1/31	1/63
63	1/31	1/63	1/127
127	1/63	1/127	1/255
255	1/127	1/255	−100

**Table 6:** Updated Q-table as a result of applying Equation (7).

	BE − 1	BE	BE + 1
3	−100	−0.28667	−0.46571
7	1/3	1/7	1/15
15	1/7	1/15	1/31
31	1/15	1/31	1/63
63	1/31	1/63	1/127
127	1/63	1/127	1/255
255	1/127	1/255	−100

## Data Availability

The data presented in this study are available on request from the first author.
